# Examining selection in Affordable Care Act (ACA) Marketplaces: special enrollment periods

**DOI:** 10.1093/haschl/qxaf048

**Published:** 2025-03-07

**Authors:** Saumya Chatrath, Alison A Galbraith, Laura F Garabedian

**Affiliations:** Department of Population Medicine, Harvard Medical School and Harvard Pilgrim Health Care Institute, Boston, MA 02215, United States; Department of Population Medicine, Harvard Medical School and Harvard Pilgrim Health Care Institute, Boston, MA 02215, United States; Department of Pediatrics, Boston Medical Center and Boston University Chobanian & Avedisian School of Medicine, Boston, MA 02118, United States; Department of Population Medicine, Harvard Medical School and Harvard Pilgrim Health Care Institute, Boston, MA 02215, United States

**Keywords:** Marketplace, special enrollment period, adverse selection

## Abstract

**Introduction:**

Affordable Care Act (ACA) Marketplace members who enroll through a special enrollment period (SEP) have significantly higher average monthly spending than members who enroll through the annual open enrollment period (OEP), driven primarily by higher inpatient spending.

**Methods:**

Using data from a large national insurer that participated in the federal ACA Marketplace from 2015 to 2016 in 24 US states, we examined differences between SEP and OEP Marketplace enrollees in time from enrollment to inpatient use of predictable and discretionary care (ie, hip and knee replacement), predictable and nondiscretionary care (ie, childbirth), and nonpredictable and nondiscretionary care (ie, acute myocardial infarction and stroke). We examined whether a 2016 policy that increased SEP eligibility verification requirements was associated with changes in utilization.

**Results:**

When compared with OEP Marketplace members, SEP members had significantly higher rates of care in all 3 categories. The 2016 policy was not associated with changes in utilization rates.

**Conclusion:**

Our results provide evidence that there is adverse selection in the SEP of the ACA Marketplaces. However, since SEP members were more likely to seek care for services that are predictable and nonpredictable, and discretionary and nondiscretionary, the optimal policy response to reduce adverse selection needs to be nuanced and multipronged.

## Introduction

Adverse selection, the phenomenon in which individuals are more likely to seek out health insurance coverage when they have medical needs,^1,2^ threatens the stability of health insurance markets.^3,4^ Affordable Care Act (ACA) Marketplaces were designed with a number of policies to deter potential adverse selection. Inducements, such as premium tax credits and cost-sharing reductions, and financial penalties for failing to obtain coverage (which were removed in 2019) were implemented to encourage healthy people to also enroll.^5-7^ Risk-adjustment and reinsurance programs were created to deter insurers from “cherry picking” healthier members and to compensate plans with sicker-on-average members.^8^ Finally, to deter people from enrolling in response to a medical need, Marketplace plans have a limited annual open enrollment period (OEP), which typically runs from November to January. Individuals (and families) can enroll outside of the annual OEP, via a special enrollment period (SEP), only if they have a “qualifying” life event, which includes changes in insurance coverage (eg, loss of employer-sponsored insurance or Medicaid), household (ie, marriage, birth, death), residence, and citizenship.^9^

Despite these measures, there is evidence of potential adverse selection among Marketplace SEP enrollees. In prior work,^10^ we found that Marketplace members who enrolled through the SEP had significantly higher average monthly spending as compared with those who enrolled through the OEP, driven primarily by higher all-cause inpatient and emergency department (ED) spending. Other researchers have found that fewer than 15% of SEP-eligible members sign up for coverage in the Marketplace,^11^ and that risk adjustment does not adequately compensate for SEP members.^12^ The higher-than-expected costs of SEP members are borne by the insurers, who will compensate by raising health insurance premiums for the Marketplace plans or exiting the Marketplace altogether.^3,13^ Therefore, the higher costs among SEP members may lead to higher Marketplace premiums and less consumer choice.

Understanding why SEP members are higher cost than OEP members will inform the optimal policy response to improve the functioning of the Marketplace. In response to insurers’ concerns about adverse selection among SEP members,^14^ the federal government implemented stricter SEP verification policies for federally run Marketplaces starting in July 2016.^15^ Prior to 2016, members could enroll in the Marketplace through an SEP based solely on a self-attestation of a qualifying event.^16^ In 2016, the Centers for Medicare and Medicaid Services (CMS) implemented a requirement that members must submit paperwork to verify their SEP-qualifying event.^14^ If consumers were unable to prove their SEP eligibility, then their coverage would be terminated. The CMS estimated there was a 20% reduction in the number of Marketplace SEP enrollees in July–December 2016 as compared with the same time period in 2015,^17^ but the impact of this policy on health care utilization has not been studied. This policy was implemented to discourage SEP-ineligible people from obtaining coverage through the SEP in response to anticipated health care needs. But increasing the administrative burden for SEP-eligible people could also deter healthy people from enrolling. A priori, it was unclear if these documentation requirements would improve the Marketplace risk pool or exacerbate adverse selection. Conversely, more recent Marketplace policies have sought to reduce administrative burden for SEP enrollment, including the “COVID-19 SEP” during the pandemic^18,19^ and the “Medicaid Unwinding SEP” after the end of the COVID-19 Public Health Emergency,^19,20^ which removed Marketplace SEP-qualifying-event verification requirements.

In this study, we examined differences between SEP and OEP enrollees in time from enrollment to multiple different categories of inpatient utilization to explore whether the higher costs among SEP enrollees are driven primarily by discretionary and predictable care or are reflective of a more vulnerable population with greater health needs requiring nondiscretionary and/or nonpredictable care. We then evaluated changes in utilization associated with the 2016 SEP-qualifying-even verification policy on medical utilization of SEP Marketplace members.

## Data and methods

### Data and study population

We used member-level administrative and health claims data from Optum's de-identified Clinformatics® Data Mart Database. Our sample included members under the age of 65 years who enrolled in a Marketplace plan offered by a large national insurer in 2015 and 2016. Our study population included members enrolled in federally run Marketplace plans (ie, used the federal Healthcare.gov platform and enrollment rules) in 16 states in 2015 and in 24 states in 2016 (see Appendix Table S1 for a full list of states). Member data included information on enrollment dates and all medical and pharmacy claims. We classified members as enrolling during the OEP or SEP based on the date that their coverage began. If a member's insurance coverage began between January 1 and March 1, they were classified as enrolling during the OEP; otherwise, they were classified as enrolling via the SEP. (See Appendix Table S2 for a detailed explanation of OEP and SEP sign-up windows and member attribution to each group.) Marketplace coverage for both groups ends on December 31 of each calendar year and must be renewed annually. We included members in their first Marketplace enrollment year during the study period. In the analyses of the 2016 verification policy, we focused on SEP members who enrolled from July–December in 2015 and July–December 2016 (since the policy was implemented in July 2016).

### Measures

We used diagnosis and procedure codes to capture member-level inpatient utilization in 4 separate categories of care that vary along the dimensions of predictability and patient discretion, using a set of representative procedures listed in Table 1.^21,22^ We focused our analysis on inpatient care because SEP member inpatient costs were between 69% and 114% higher as compared with OEP members, while ED costs were only 11%–19% higher.^10^ We analyze 4 categories of care: (1) predictable and discretionary care (eg, knee and hip replacements), (2) predictable and nondiscretionary care (eg, childbirth), (3) nonpredictable and nondiscretionary care (eg, acute myocardial infarction [AMI] and stroke), and (4) nonpredictable and nondiscretionary care (eg, appendectomy). While category 3 (eg, AMI and stroke) are acute events that are largely nonpredictable in terms of timing, both events have known risk factors (eg, high blood pressure, high cholesterol, obesity). Therefore, category 3 signals members who are sicker on average who could benefit from preventive care. In contrast, category 4 (eg, appendectomy) is the closest possible medical procedure to a “random” inpatient event. Since we should not observe any differences between the groups in the use of inpatient services for this procedure, category 4 serves as a falsification test. (See Appendix Table S3 for the list of diagnosis and procedure codes used to classify the 4 categories of care.) We also examined demographic characteristics (eg, age and sex), enrollment length (number of months enrolled in the Marketplace in that calendar year), and plan characteristics (eg, health maintenance organization [HMO] or high-deductible health plan with health savings account [HDHP-HSA]).

**Table 1.** Care classification.

	Category	Procedures
Category 1	Predictable and discretionary	Hip and knee replacement
Category 2	Predictable and nondiscretionary	Childbirth
Category 3	Nonpredictable and nondiscretionary	AMI and stroke
Category 4	Falsification	Appendectomy

This table lists the reasons for inpatient hospitalizations that are used for each category. A full list of diagnosis and procedure codes used is available in Appendix Table S3.

Abbreviation: AMI, acute myocardial infarction.

### Statistical methods

First, we examined differences between OEP and SEP Marketplace members with respect to observable demographics (ie, age, sex), plan characteristics (eg, HDHP), and monthly spending per member (eg, overall, inpatient, ED, outpatient, and prescription drugs) in each study year, using *t* tests for continuous variables and chi-square tests for categorical variables.

Next, we evaluated differences in health care utilization between OEP and SEP Marketplace members. For each of the 4 categories of utilization, we used survival analysis methods to measure time from Marketplace enrollment to hospitalization within the calendar year of enrollment for SEP vs OEP members. Survival methods are preferred since, by definition, SEP and OEP members have different lengths of Marketplace enrollment within a calendar year, and the measure of time-to-utilization after enrollment is key for determining adverse selection (ie, testing whether 1 group was systematically more likely to enroll in anticipation of needing services). We used Kaplan-Meier curves, with a log-rank test, to compare the cumulative incidence of inpatient use, by category, for SEP vs OEP members, separately in 2015 and 2016. We then used Cox proportional hazards regression models to estimate the hazard ratio (which we refer to as “rate”) of each utilization category for SEP vs OEP enrollees in each study year. For the Cox models, we censored members either at the date of disenrollment from the Marketplace plan or at the end of the calendar year (whichever came first). We used martingale residuals to test the proportional hazards assumption.^23,24^ In the results below, we report both unadjusted and adjusted Cox regression results (ie, controlling for age and sex). The adjusted models estimate whether there continue to be differences in utilization between SEP and OEP members after controlling for observed differences in demographic characteristics that are known to insurers at the time of SEP Marketplace enrollment. Since all risk-adjustment models use demographic adjustments, the adjusted results are closer to the results that insurers would observe. In contrast, the unadjusted results capture the actual differences in utilization and costs among SEP vs OEP members.

In the second part of the analysis, we measured the association between the July 2016 SEP-qualifying-event verification policy and changes in utilization by comparing inpatient use of SEP members who enrolled before (July–December 2015) and after (July–December in 2016) the policy change vs OEP enrollees in each year (ie, pre-post with comparison design). Since we compare the rates across years, we restricted this analysis to the 16 states that appeared in the data in both 2015 and 2016. We repeated the Cox proportional hazard regressions comparing SEP with OEP members described above but limited to SEP members enrolled from July–December in 2015 and 2016 and compare the hazard ratios and the 95% confidence intervals (CIs) in 2015 (pre-policy) and 2016 (post-policy).

## Results

Our sample included just under 1.5 million unique Marketplace members in plans offered by a large national insurer in 2015 (*n* = 732 641) and 2016 (*n* = 722 307). In both study years, OEP members were older than SEP members (41.1 vs 36.6 years, *P* < .01, in 2015; 40.1 vs 33.8 years, *P* < .01, in 2016) (Table 2). There was no difference between the 2 groups with respect to the percentage of women enrolled. Open enrollment period members were more likely to be enrolled in an HDHP-HSA in 2015 (17.7% vs 13.9%; *P* < 0.01), but the difference between the 2 narrowed in 2016 (21.5% vs 19.7%; *P* < 0.01).

**Table 2.** Demographic characteristics and monthly spending of Marketplace enrollees.

	2015			2016		
	OEP	SEP	Difference	OEP	SEP	Difference
Number of members	552 417	180 224		588 815	133 492	
Mean days enrolled	242.10	137.00	105.00∗	278.2	146.0	132.3∗
Mean age (y)	41.06	36.63	4.43∗	40.14	33.83	6.30∗
Female (%)	55.56	55.69	−0.13	55.57	55.49	0.08
HMO (%)	66.65	65.78	0.87∗	59.74	61.16	−1.42∗
HDHP (%)	17.68	13.88	3.80∗	21.49	19.69	1.83∗
Total spending ($)	542.6	720.7	−178.1∗	574.5	760.2	−185.7∗
Inpatient ($)	147.0	244.1	−97.1∗	144.3	301.0	−156.7∗
Emergency department ($)	92.77	111.5	−18.73∗	72.72	80.35	−7.63∗
Outpatient ($)	38.51	45.66	−7.15∗	55.38	46.36	9.02∗
Prescription drug ($)	49.91	59.42	−9.51∗	75.95	76.50	−0.55

This table replicates cost estimates from Garabedian et al^10^ to test whether OEP and SEP members are observably similar. HMO is a binary flag that indicates whether a patient's insurance plan type was a Health Maintenance Organization. HDHP is a binary flag that indicates whether a patient's insurance plan was a high-deductible health plan with a Health Savings Account (HSA). All costs are at the average monthly level. The “Diff” column reports *t* tests of the difference in means between the 2 groups for continuous variables, such as number of days enrolled and age. For binary variables, such as female, we report the chi-square test results. *P < .01.

Abbreviations: OEP, open enrollment period; SEP, special enrollment period.

### Utilization differences between SEP and OEP Marketplace members

Compared with OEP members, SEP members were more likely to be hospitalized for an inpatient procedure for 3 of the 4 categories of care studied. As demonstrated in the Kaplan-Meier cumulative incidence curves (Appendix Figure S1), SEP members (orange) had a higher rate of utilization during Marketplace enrollment as compared with OEP members (blue) for care that is (1) predictable and discretionary, (2) predictable and nondiscretionary, and (3) nonpredictable and nondiscretionary. Appendix Table S4 includes the results for the log-rank test statistics and the corresponding *P* values. The Cox regression model results in Table 3 corroborate the Kaplan-Meier curves. The hazard ratios for predictable and discretionary inpatient care (eg, hip and knee replacement) for SEP vs OEP enrollees were 1.59 in 2015 (95% CI: 1.39, 1.82) and 1.40 in 2016 (95% CI: 1.22, 1.61). This means that SEP enrollees had 1.6 times the rate of hip and knee replacements than OEP enrollees in 2015 and 1.4 times the rate in 2016. The hazard ratios for predictable and nondiscretionary inpatient care (eg, childbirth) for SEP vs OEP enrollees were 1.84 in 2015 (95% CI: 1.71, 1.98) and 1.61 in 2016 (95% CI: 1.50, 1.73), and the hazard ratios for nonpredictable and nondiscretionary care (eg, AMI and stroke) for SEP vs OEP enrollees were 1.38 in 2015 (95% CI: 1.26, 1.51) and 1.52 in 2016 (95% CI: 1.37, 1.69). The results for the unadjusted Cox model (Appendix Table S5) were similar, but with smaller hazard ratios for hip and knee replacement and AMI and stroke. This means that, conditional on age and sex, SEP members were even more likely than OEP members to receive care for these categories. The results were also similar when restricting the 2016 analysis to the 16 states that the insurer offered Marketplace coverage to in both study years (Appendix Table S6). For the falsification test, there was no statistically significant difference between SEP and OEP members for the measure of nonpredictable, nondiscretionary care (eg, appendectomy) in the unadjusted or adjusted models.

**Table 3.** SEP vs OEP utilization by category, by year.

Category	Procedure	2015 (95% CI)	2016 (95% CI)
Predictable and discretionary	Hip and knee replacement	1.59 (1.39–1.82)	1.40 (1.22–1.61)
Predictable and nondiscretionary	Childbirth	1.84 (1.71–1.98)	1.61 (1.50–1.73)
Nonpredictable and nondiscretionary	AMI and stroke	1.38 (1.26–1.51)	1.52 (1.37–1.69)
Falsification	Appendectomy	1.08 (0.87–1.33)	0.90 (0.71–1.15)

The table shows the Cox regression results comparing special enrollment period (SEP) with open enrollment period (OEP) members in each year, adjusted for age and sex. Hazard ratios over 1 indicate higher utilization among SEP members.

Abbreviation: AMI, acute myocardial infarction.

### Evaluation of the 2016 SEP-qualifying-event verification policy

In the repeated survival analysis (ie, restricted to July–December 2016 SEP enrollees who were impacted by the July 2016 policy, and a pre-policy comparison of July–December 2015 enrollees), the Kaplan-Meier incidence curves (Appendix Figure S2, Appendix Table S7) and adjusted Cox regression results (Table 4) demonstrate that SEP members were more likely to incur care than OEP members across all 3 categories in 2015. Even after the eligibility verification policy was implemented in 2016, we continue to see the same pattern (ie, SEP members continue to be more likely to receive inpatient care for the categories identified). The hazard ratios for predictable and discretionary care (eg, hip and knee replacement) were 1.65 (95% CI: 1.34, 2.02) in 2015 and 1.53 (95% CI: 1.21, 1.95) in 2016. The hazard ratios for nonpredictable and nondiscretionary care (eg, AMI and stroke) were 1.43 (95% CI: 1.25, 1.63) in 2015 and 1.41 (95% CI: 1.18, 1.70) in 2016. The hazard ratios for predictable and nondiscretionary care (eg, childbirth) declined after the policy, from 2.05 (95% CI: 1.83, 2.29) in 2015 to 1.68 in 2016 (95% CI: 1.48, 1.91), but the 95% CIs overlap. Results were similar in the unadjusted models (Appendix Table S8).

**Table 4.** SEP vs OEP utilization by category before (2015) and after (2016) the SEP-qualifying-event verification requirement.

Category	Procedure	2015 (95% CI)	2016 (95% CI)
Predictable and discretionary	Hip and knee replacement	1.65 (1.34–2.02)	1.53 (1.21–1.95)
Predictable and nondiscretionary	Childbirth	2.05 (1.83–2.29)	1.68 (1.48–1.91)
Nonpredictable and nondiscretionary	AMI and stroke	1.43 (1.25–1.63)	1.41 (1.18–1.70)
Falsification	Appendectomy	1.13 (0.83–1.54)	0.79 (0.51–1.23)

The table shows the Cox regression results comparing special enrollment period (SEP) with open enrollment period (OEP) members, adjusted for age and sex, before and after the July 2016 SEP qualifying-event verification policy change went into effect. The 2015 results compare July–December SEP vs OEP enrollees in 2015, and the 2016 results compare July–December SEP vs OEP enrollees in 2016. This analysis is restricted to members in the 16 states that the insurer offered Marketplace coverage to in both 2015 and 2016. Hazard ratios over 1 indicate higher utilization among SEP members.

Abbreviation: AMI, acute myocardial infarction.

## Discussion

There are 2 broad takeaways from our study on Marketplace SEPs. One, Marketplace members who enroll during an SEP were more likely to be hospitalized as compared with their OEP counterparts for care spanning the dimensions of predictability and discretion. This suggests that SEP patients are both sicker on average, and also more likely to seek out discretionary care. Second, a policy to increase administrative barriers to Marketplace SEP enrollment, with the explicit focus to reduce adverse selection among SEP enrollees, did not achieve its policy aim of reducing care that is predictable and discretionary (eg, hip and knee replacement).

The SEP enrollees were more likely than OEP enrollees to incur inpatient care across 3 categories that we identified: predictable and discretionary (eg, knee and hip replacement), predictable and nondiscretionary (eg, childbirth), and nonpredictable and nondiscretionary (eg, myocardial infarction and stroke). Since we found evidence that the SEP Marketplace population was adversely selected across all 3 categories, it is unclear what the optimal policy response should be to reduce adverse selection.

For example, we selected hip and knee replacements to examine care that is predictable and discretionary. These procedures are typically scheduled far in advance, and they are usually nonemergent so patients can choose when they want to undergo them. This is a classic example of the type of selection that policymakers and insurers would like to discourage because it implies that patients sign up for Marketplace coverage through the SEP with the explicit purpose of receiving a procedure. As a consequence, the average costs in the Marketplace increase and this puts upward pressure on premiums in subsequent years. We found that SEP members are approximately 1.4 times more likely to be hospitalized for hip and knee replacements as compared with OEP members in 2016. If this was the only type of selection that we observed, then the solution would be straightforward: enacting barriers to enrollment will prevent patients with discretionary health needs from signing up for the explicit purpose of covering expensive, elective surgeries. There is, however, another type of adverse selection at play here; patients with nondiscretionary health care needs also obtain Marketplace coverage via the SEP. The Marketplaces were established to broaden access to health care coverage and deterring these patients from enrolling would go against the stated aims of the ACA.^25^ We found that, compared with OEP members, SEP members were about twice as likely to receive inpatient care for childbirth. We also found that, compared with OEP members, SEP members were more likely to have AMI or stroke, indicating they are sicker on average with risk factors that contribute to these conditions. These are health care needs that cannot be deferred or timed with the open enrollment season and may make these individuals more likely to pursue coverage via an SEP compared with their healthier counterparts.

Since Marketplace SEP enrollees have higher costs than OEP enrollees, insurers are more likely to incur losses from SEP members. Facing financial losses in the Marketplace, insurers might choose to increase premiums the following year or exit the individual exchanges, as United Healthcare did in 2017.^3^ Increased premiums would increase the cost of health insurance for unsubsidized Marketplace enrollees but could actually reduce the cost of health insurance among Marketplace enrollees who are eligible for premium subsidies (since the subsidies are tied to price),^26^ which would increase premium subsidy amounts paid by the federal government.

There are 3 possible options that can be used by policymakers to try to address insurer concerns about SEP adverse selection in the Marketplace. First, they could try to limit enrollment in the SEP by enacting stricter requirements for signing up for coverage (eg, a more stringent qualifying-event verification process as they did in 2016). Second, they can try to better compensate insurers for sicker SEP enrollees via mechanisms such as risk adjustment or reinsurance.^27,28^ Prior research has found that risk-adjustment algorithms underpay for SEP members^12^ and new risk-adjustment rules that better account for enrollment duration (ie, partial-year enrollees) were implemented in 2023.^29^ If insurers are adequately compensated for these enrollees (ie, SEP enrollees are profitable), they would not be as concerned about the higher costs of SEP enrollees in their plans. Third, policymakers could induce more young and healthy people to sign up for coverage when they become SEP-eligible and thus expand the risk pool and reduce average per-member costs. It is estimated that only 15% of SEP-eligible patients signed up for Marketplace coverage via the SEP.^11^ People would be more likely to sign up if there are reduced frictions to sign up, and if coverage is affordable.^30^ Other interventions, such as targeted telephone outreach, could also increase recruitment of SEP-eligible members, particularly vulnerable subgroups.^31^

The introduction of the qualifying-event eligibility verification requirements in 2016 was an attempt to remedy adverse selection by deterring enrollees with predictable health needs from enrolling in the Marketplace via the SEP. This policy prohibits SEP-ineligible patients from enrolling in the Marketplace, and also likely deters SEP-eligible patients due to administrative barriers. Since this policy is indiscriminate, it could reduce adverse selection by deterring enrollment among people seeking predictable/discretionary care and also sicker-on-average and more vulnerable populations who need nonpredictable/nondiscretionary care. We found that the policy was not associated with changes in utilization for AMI and stroke (ie, nonpredictable and nondiscretionary) or knee and hip replacements (ie, predictable and discretionary). Furthermore, this policy could potentially exacerbate adverse selection if the administrative burden disproportionately discourages eligible healthy people from signing up. Prior research among Medicaid patients and Supplemental Nutrition Assistance Program (SNAP) recipients has found that increased administrative burden reduces enrollment in public programs for eligible individuals.^32,33,34^ A study of Massachusetts’ subsidized exchange, a precursor to the ACA Marketplaces, found that increased administrative burden reduced enrollment and disproportionately deterred younger, healthier, and lower-income people from enrolling.”^35^

Pregnant people are a vulnerable population that may be particularly impacted by SEP-qualifying-event eligibility verification rules. SEP enrollees were more likely than OEP enrollees to be hospitalized for childbirth, suggesting that SEPs are an important contributor to Marketplace coverage of pregnant people. Although not statistically significant, the 2016 verification policy reduced the rate of childbirth among SEP enrollees by a large magnitude. This likely means that the verification requirements and/or paperwork burden discouraged pregnant people, a population with genuine nondeferrable health care needs, from obtaining Marketplace coverage. Prior research has shown that nearly 40% of pregnant women with short-term enrollment in Marketplace plans report being uninsured before Marketplace enrollment.^36^ In post hoc analysis (Appendix Figure S3) we found that pregnant SEP members enrolled later in their pregnancy than pregnant OEP enrollees. This is troublesome given that health insurance coverage is vital for access to affordable, high-quality, timely prenatal care.^37,38^ Pregnant people who have continuous enrollment in a Marketplace plan from pre-conception through the postpartum period have a higher rate of timely and adequate prenatal care, compared with pregnant people who enrolled in the Marketplace later in their pregnancy.^36^ While childbirth is an SEP-qualifying event, pregnancy is not. In recent years, 8 US states and Washington, DC, with state-based Marketplaces—starting with New York in 2016—have enacted policies that include pregnancy as an SEP-qualifying event for Marketplace coverage, meaning that pregnant people in these states can enroll in a Marketplace plan when they become pregnant.^39^ This policy increased Marketplace coverage among eligible pregnant people in New York.^39^ Future research should evaluate the impact of allowing pregnancy as an SEP-qualifying event on access to timely prenatal care and improved infant and maternal health outcomes.

Our study has several limitations. Our results represent the experience of 1 large, national insurer that participates in federal Marketplaces in 24 states. However, our results may not reflect changes among Marketplace SEP enrollees in other health plans or in state-based Marketplaces. Since we have data from only 1 insurer, we are unable to determine the prior insurance status of Marketplace members in our study. We also do not have information on whether Marketplace members had an SEP-qualifying event. While we cannot observe whether the qualifying-event requirements deterred ineligible patients from signing up, we can observe if there is a change in the SEP risk pool. The insurer entered the Marketplace in 2015 and chose not to participate in the Marketplaces in most states starting in 2017, which limits the study period to only 2 full years of data in 2015–2016. Since we only have 1 year of data pre-policy, we cannot compare pre-policy trends. Finally, we studied a limited list of representative procedures for each category of care, and did not explicitly examine the cost associated with this care. We made assumptions about whether each procedure type was discretionary or predictable and may have misclassified certain episodes of care (eg, a hip replacement surgery after a hip fracture is not predictable and likely not discretionary). We chose procedures that were relatively common among the Marketplace population so that we had adequate study power, but also so that we could examine large drivers of inpatient costs. We did not adjust for multiple testing. Finally, some of our results could be driven by seasonality of health care outcomes, particularly AMI (more common in winter) and childbirth (more common in summer/fall). However, we did not control for seasonality in our analyses since the SEP is also seasonal, and we wanted to explain why the SEP enrollees are higher cost. Future research and risk-adjustment algorithm development should consider the interplay between seasonality of health outcomes and Marketplace enrollment rules.

## Conclusion

Our results provide evidence that there are different types of adverse selection occurring in the Marketplace SEPs. Compared with patients who obtain coverage via the OEP, patients who enrolled via SEPs sought more medical care for nondeferrable health care needs and also discretionary care. It is difficult to target policies to reduce adverse selection from predictable/discretionary care while also ensuring that sicker-than-average populations and pregnant people with nondiscretionary health needs have access to coverage. We also found that a policy to increase qualifying-event verification requirements for SEP enrollment was not associated with changes in utilization for most of the categories we studied, but may have reduced SEP Marketplace coverage for pregnant people. Our results have implications for recent policies that facilitated access to the Marketplace by removing the SEP-qualifying-event verification process (eg, COVID-19 SEPs, Medicaid Unwinding SEP) and future policies that seek to deter access to Marketplace coverage by enhancing qualifying-event verification requirements.^40^

## Supplementary Material

qxaf048_Supplementary_Data
