# Supplementary material for: Examining selection in Affordable Care Act (ACA) Marketplaces: special enrollment periods
Source: Health Aff Sch. 2025 Mar 7;3(4):qxaf048. doi: 10.1093/haschl/qxaf048 (PMC11976056; doi:10.1093/haschl/qxaf048)
Supplement: qxaf048_Supplementary_Data [file qxaf048_supplementary_data.zip › Marketplace SEP Appendix 2.24.25.docx]

**Appendix**

Supplement to: Chatrath S, Galbraith A, Garabedian LF. *Examining Selection in Affordable Care Act (ACA) Marketplaces: Special Enrollment Periods*

**Table S1**: List of States Participating in Federally-run Exchanges Included in Study

**Table S2**: Member Attribution – Timing of Marketplace Enrollment

**Table S3**: Diagnosis and Procedure Codes for Each Category of Care

**Figure S1:** Unadjusted Kaplan Meier Cumulative Incidence Curves: Special Enrollment Period (SEP) vs. Open Enrollment Period (OEP)

**Table S4.** Log Rank Results - Full Year

**Table S5**: Utilization among Marketplace Members who Enrolled in the Special Enrollment Period (SEP) vs. Open Enrollment Period (OEP) in 2015 and 2016 (unadjusted hazard ratios)

**Table S6**: Utilization among Marketplace Members who Enrolled in the Special Enrollment Period (SEP) vs. Open Enrollment Period (OEP) in 2015 and 2016 (adjusted hazard ratios, restricted to 2015 states)

**Figure S2:** Time from Marketplace Enrollment to Utilization among Marketplace Members who Enrolled in the Special Enrollment Period (SEP) vs. Open Enrollment Period (OEP) before (2015) and After (2016) the SEP Eligibility Verification Policy (Kaplan-Meier cumulative incidence curves)

**Table S7:** Log Rank Test - Eligibility Verification Policy

**Table S8**: Utilization among Marketplace Members who Enrolled in the Special Enrollment Period (SEP) vs. Open Enrollment Period (OEP) before (2015) and After (2016) the SEP Eligibility Verification Policy (unadjusted hazard ratios)

**Figure S3**: Distribution of Days from Marketplace Enrollment to Childbirth for Marketplace Members who Enrolled in the Open Enrollment Period (OEP) vs. Special Enrollment Period (SEP) in 2015 and 2016

Table S1: List of States Participating in Federally-run Exchanges Included in Study


| Year | Participating States |
| --- | --- |
| 2015 | AL, AZ, FL, GA, IL, IN, LA, MI, MO, MS, NC, NJ, OH, PA, TX, WI |
| 2016 | AL*, AR, AZ*, FL*, GA*, IA, IL*, IN*, KS, LA*, MI*, MO*, MS*, NC*, NE, NJ*, OH*, OK, PA*, SC, TN, TX*, VA, WI* |



Note: Stars denote the 16 states that the insurer offered Marketplace coverage to in both 2015 and 2016.

Table S2: Member Attribution – Timing of Marketplace Enrollment

| Year | Open Enrollment Sign-Up | Special Enrollment Sign-Up |
| --- | --- | --- |
| 2015 | November 15, 2014 - February 15, 2015 | After February 15, 2015 |
| 2016 | November 1, 2015 - January 31, 2016 | After January 31, 2016 |

Note: Table shows sign-up windows for OEP and SEP. The first possible Marketplace coverage start date is January 1st each year. In both study years, a member was assigned to the OEP if their Marketplace coverage started from January 1-March 1. All other coverage start dates were assigned to the SEP. Members who sign up in November to mid-December start coverage on January 1st. For those who do not start coverage on January 1st, the following rules apply. Coverage begins on the first day of the following month for members who sign up between the first and fifteenth day of the month. For those signing up between the sixteen and the end of the month, coverage begins on the first day of the second following month. For example, if a member signs up for coverage on January 14 their coverage begins on February 1, but if they sign up on January 16 their coverage begins on March 1. We can only observe enrollment start dates in our data, so we impute whether a member signed up via the OEP or SEP by using these dates.

Table S3. Diagnosis and Procedure Codes for Each Category of Care

| Category | Type of Inpatient Care | ICD-9 Codes | ICD-10 Codes | CPT Codes |
| --- | --- | --- | --- | --- |
| Predictable & Discretionary | Hip Replacements | 7910, 7915, 7930, 7935, 7855, 8151, 8152 (excluding diagnosis codes 820, 820.3, 820.31, 820.32, 820.8, 820.9) | 0PS004Z, 0PS034Z, 0PS044Z, 0PS104Z, 0PS134Z, 0PS144Z, 0PS204Z, 0PS234Z, 0PS244Z, 0PS304Z, 0PS334Z, 0PS344Z, 0PS404Z, 0PS434Z, 0PS444Z, 0PS504Z, 0PS534Z, 0PS544Z, 0PS604Z, 0PS634Z, 0PS644Z, 0PS704Z, 0PS734Z, 0PS744Z, 0PS804Z, 0PS834Z, 0PS844Z, 0PS904Z, 0PS934Z, 0PS944Z, 0PSB04Z, 0PSB34Z, 0PSB44Z, 0PSC04Z, 0PSC34Z, 0PSC44Z, 0PSD04Z, 0PSD34Z, 0PSD44Z, 0PSF04Z, 0PSF34Z, 0PSF44Z, 0PSG04Z, 0PSG34Z, 0PSG44Z, 0PSH04Z, 0PSH34Z, 0PSH44Z, 0PSJ04Z, 0PSJ34Z, 0PSJ44Z, 0PSK04Z, 0PSK34Z, 0PSK44Z, 0PSL04Z, 0PSL34Z, 0PSL44Z, 0PSM04Z, 0PSM34Z, 0PSM44Z, 0PSN04Z, 0PSN34Z, 0PSN44Z, 0PSP04Z, 0PSP34Z, 0PSP44Z, 0PSQ04Z, 0PSQ34Z, 0PSQ44Z, 0PST04Z, 0PST34Z, 0PST44Z, 0PSV04Z, 0PSV34Z, 0PSV44Z, 0QH604Z, 0QH606Z, 0QH634Z, 0QH636Z, 0QH644Z, 0QH646Z, 0QH704Z, 0QH706Z, 0QH734Z, 0QH736Z, 0QH744Z, 0QH746Z, 0QH804Z, 0QH806Z, 0QH834Z, 0QH836Z, 0QH844Z, 0QH846Z, 0QH904Z, 0QH906Z, 0QH934Z, 0QH936Z, 0QH944Z, 0QH946Z, 0QHB04Z, 0QHB06Z, 0QHB34Z, 0QHB36Z, 0QHB44Z, 0QHB46Z, 0QHC04Z, 0QHC06Z, 0QHC34Z, 0QHC36Z, 0QHC44Z, 0QHC46Z, 0QS004Z, 0QS034Z, 0QS044Z, 0QS204Z, 0QS234Z, 0QS244Z, 0QS304Z, 0QS334Z, 0QS344Z, 0QS404Z, 0QS434Z, 0QS444Z, 0QS504Z, 0QS534Z, 0QS544Z, 0QS604Z, 0QS604Z, 0QS606Z, 0QS634Z, 0QS634Z, 0QS636Z, 0QS644Z, 0QS646Z, 0QS704Z, 0QS706Z, 0QS734Z, 0QS736Z, 0QS744Z, 0QS746Z, 0QS804Z, 0QS806Z, 0QS834Z, 0QS836Z, 0QS844Z, 0QS846Z, 0QS904Z, 0QS906Z, 0QS934Z, 0QS934Z, 0QS936Z, 0QS944Z, 0QS946Z, 0QSB04Z, 0QSB06Z, 0QSB34Z, 0QSB34Z, 0QSB36Z, 0QSB44Z, 0QSB46Z, 0QSC04Z, 0QSC06Z, 0QSC34Z, 0QSC36Z, 0QSC44Z, 0QSC46Z, 0QSD04Z, 0QSD34Z, 0QSD44Z, 0QSF04Z, 0QSF34Z, 0QSF44Z, 0QSG04Z, 0QSG34Z, 0QSG44Z, 0QSH04Z, 0QSH34Z, 0QSH44Z, 0QSJ04Z, 0QSJ34Z, 0QSJ44Z, 0QSK04Z, 0QSK34Z, 0QSK44Z, 0QSL04Z, 0QSL34Z, 0QSL44Z, 0QSM04Z, 0QSM34Z, 0QSM44Z, 0QSN042, 0QSN04Z, 0QSN342, 0QSN34Z, 0QSN442, 0QSN44Z, 0QSP042, 0QSP04Z, 0QSP342, 0QSP34Z, 0QSP442, 0QSP44Z, 0QSQ04Z, 0QSQ34Z, 0QSQ44Z, 0QSR04Z, 0QSR34Z, 0QSR44Z, 0SR9019, 0SR901A, 0SR901Z, 0SR9029, 0SR902A, 0SR902Z, 0SR9039, 0SR9039, 0SR903A, 0SR903Z, 0SR9049, 0SR904A, 0SR904Z, 0SR9069, 0SR906A, 0SR906Z, 0SR907Z, 0SR90J9, 0SR90JA, 0SR90JZ, 0SR90KZ, 0SRA009, 0SRA00A, 0SRA00Z, 0SRA019, 0SRA01A, 0SRA01Z, 0SRA039, 0SRA03A, 0SRA03Z, 0SRA07Z, 0SRA0J9, 0SRA0JA, 0SRA0JZ, 0SRA0KZ, 0SRB019, 0SRB01A, 0SRB01Z, 0SRB029, 0SRB02A, 0SRB02Z, 0SRB039, 0SRB03A, 0SRB03Z, 0SRB049, 0SRB04A, 0SRB04Z, 0SRB069, 0SRB06A, 0SRB06Z, 0SRB07Z, 0SRB0J9, 0SRB0JA, 0SRB0JZ, 0SRB0KZ, 0SRE009, 0SRE00A, 0SRE00Z, 0SRE019, 0SRE01A, 0SRE01Z, 0SRE039, 0SRE03A, 0SRE03Z, 0SRE07Z, 0SRE0J9, 0SRE0JA, 0SRE0JZ, 0SRE0KZ, 0SRR019, 0SRR01A, 0SRR01Z, 0SRR039, 0SRR03A, 0SRR03Z, 0SRR07Z, 0SRR0J9, 0SRR0JA, 0SRR0JZ, 0SRR0KZ, 0SRS019, 0SRS01A, 0SRS01Z, 0SRS039, 0SRS03A, 0SRS03Z, 0SRS07Z, 0SRS0J9, 0SRS0JA, 0SRS0JZ, 0SRS0KZ (excluding XYZ) | 27130, 27132, 27134, 27137, 27138 |
| Predictable & Discretionary | Knee Replacement | 0080, 0081, 0082, 0083, 0084, 8154, 8155 | 0QPD0JZ, 0QPD3JZ, 0QPD4JZ, 0QPF0JZ, 0QPF3JZ, 0QPF4JZ, 0QRD0JZ, 0QRD3JZ, 0QRD4JZ, 0QRF0JZ, 0QRF3JZ, 0QRF4JZ, 0QUD0JZ, 0QUD3JZ, 0QUD4JZ, 0QUF0JZ, 0QUF3JZ, 0QUF4JZ, 0SPC08Z, 0SPC09Z, 0SPC0JZ, 0SPC48Z, 0SPC48Z, 0SPC48Z, 0SPC4JZ, 0SPC4JZ, 0SPC4JZ, 0SPD08Z, 0SPD08Z, 0SPD08Z, 0SPD09Z, 0SPD0JZ, 0SPD48Z, 0SPD4JZ, 0SPT0JZ, 0SPU0JZ, 0SPW0JZ, 0SRC069, 0SRC06A, 0SRC06Z, 0SRC0J9, 0SRC0JA, 0SRC0JZ, 0SRD069, 0SRD06A, 0SRD06Z, 0SRD0J9, 0SRD0JA, 0SRD0JZ, 0SRT0J9, 0SRT0JA, 0SRT0JZ, 0SRU0J9, 0SRU0JA, 0SRU0JZ, 0SRV0J9, 0SRV0JA, 0SRV0JZ, 0SRW0J9, 0SRW0JA, 0SRW0JZ, 0SUC09C, 0SUV09Z, 0SUW09Z | 27445, 27446, 27447, 27486, 27487 |
| Predictable & Non-Discretionary | Childbirth | 6662, 6901, 6951, 6952, 720, 721, 7221, 7229, 7231, 7239, 724, 7251, 7252, 7253, 7254, 726, 7271, 7279, 728, 729, 7301, 7309, 731, 7321, 7322, 733, 734, 7351, 7359, 736, 738, 7391, 7392, 7393, 7394, 7399, 740, 741, 742, 744, 7499 | 0Q820ZZ, 0Q823ZZ, 0Q824ZZ, 0Q830ZZ, 0Q833ZZ, 0Q834ZZ, 0U7C7ZZ, 0UB50ZZ, 0UB53ZZ, 0UB54ZZ, 0UB57ZZ, 0UB58ZZ, 0UB60ZZ, 0UB63ZZ, 0UB64ZZ, 0UB67ZZ, 0UB68ZZ, 0UT50ZZ, 0UT54ZZ, 0UT60ZZ, 0UT64ZZ, 0W8NXZZ, 10900ZC, 10903ZC, 10904ZC, 10907ZA, 10907ZC, 10908ZA, 10A07Z6, 10A07ZZ, 10A08ZZ, 10D00Z0, 10D00Z1, 10D00Z2, 10D07Z3, 10D07Z4, 10D07Z5, 10D07Z6, 10D07Z7, 10D07Z8, 10D17ZZ, 10D18ZZ, 10E0XZZ |  |
| Non-Predictable & Non-Discretionary | Acute Myocardial Infarction (AMI) | 41001, 41011, 41021, 41031, 41041, 41051, 41061, 41071, 41081, 41091 | I21, I210, I2101, I2102, I2109, I211, I2111, I2119, I212, I2121, I2129, I213, I214, I219, I21A1, I21A9, I22, I220, I221, I222, I228, I229 |  |
| Non-Predictable & Non-Discretionary | Stroke | 430, 431, 436, 43301, 43311, 43321, 43331, 43381, 43391, 43401, 43411, 43491 | I60, I600, I6000, I6001, I6002, I601, I6010, I6011, I6012, I602, I6020, I6021, I6022, I603, I6030, I6031, I6032, I604, I605, I6050, I6051, I6052, I606, I607, I608, I609, I61, I610, I611, I612, I613, I614, I615, I616, I618, I619, I63, I630, I6300, I6301, I63011, I63012, I63013, I63019, I6302, I6303, I63031, I63032, I63033, I63039, I6309, I631, I6310, I6311, I63111, I63112, I63113, I63119, I6312, I6313, I63131, I63132, I63133, I63139, I6319, I632, I6320, I6321, I63211, I63212, I63213, I63219, I6322, I6323, I63231, I63232, I63233, I63239, I6329, I633, I6330, I6331, I63311, I63312, I63313, I63319, I6332, I63321, I63322, I63323, I63329, I6333, I63331, I63332, I63333, I63339, I6334, I63341, I63342, I63343, I63349, I6339, I634, I6340, I6341, I63411, I63412, I63413, I63419, I6342, I63421, I63422, I63423, I63429, I6343, I63431, I63432, I63433, I63439, I6344, I63441, I63442, I63443, I63449, I6349, I635, I6350, I6351, I63511, I63512, I63513, I63519, I6352, I63521, I63522, I63523, I63529, I6353, I63531, I63532, I63533, I63539, I6354, I63541, I63542, I63543, I63549, I6359, I638, I639 |  |
| Falsification | Appendectomy | 470, 4701, 4709 | 0DTJ4ZZ, 0DTJ0ZZ, 0DTJ7ZZ, 0DTJ8ZZ | 44950, 44955, 44960, 44970 |

Figure S1**:** Unadjusted Kaplan-Meier Cumulative Incidence Curves: Special Enrollment Period (SEP) vs. Open Enrollment Period (OEP)

Predictable & Discretionary [Hip & Knee Replacement]
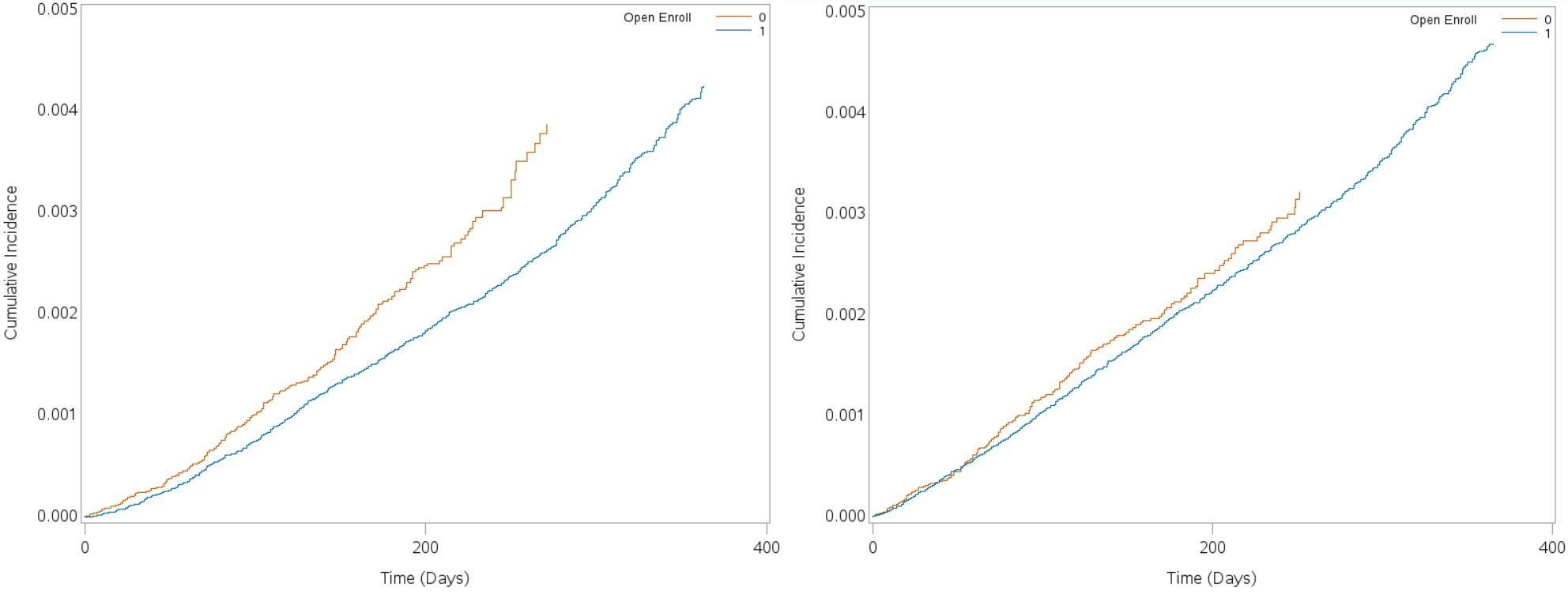


(a) 2015 (b) 2016

Predictable & Non-Discretionary [Childbirth]
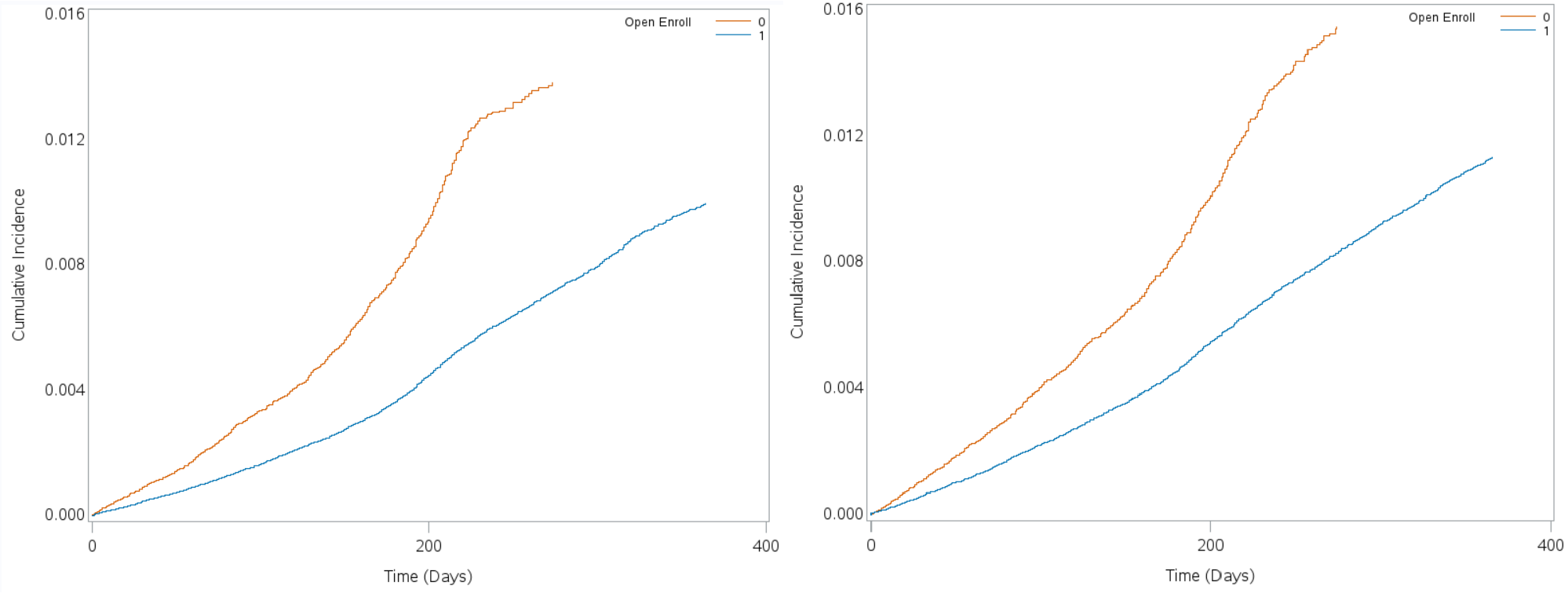


(c) 2015 (d) 2016

Non-Predictable & Non-Discretionary [AMI & Stroke]
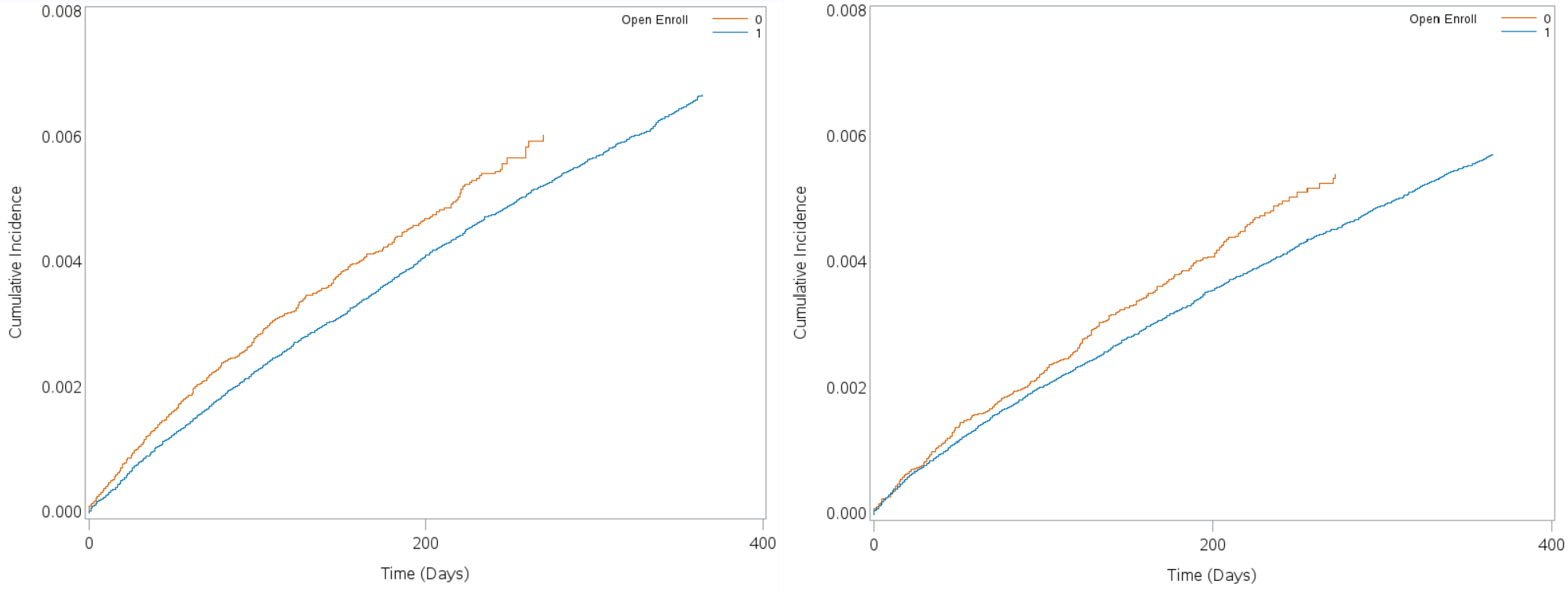


(e) 2015 (f) 2016

Predictable & Non-Discretionary [Appendectomy – Falsification Test]


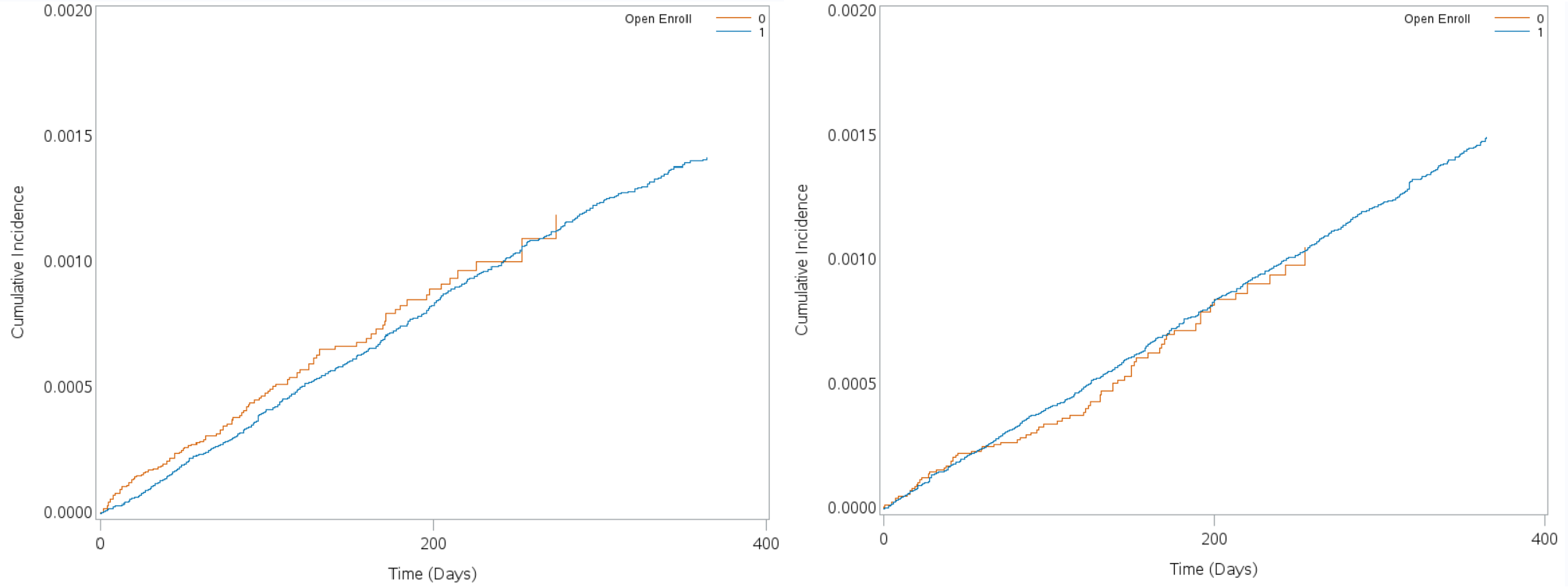


(g) 2015 (h) 2016

Note: Figures show the Kaplan-Meier cumulative incidence curves for SEP (orange) and OEP (blue) members.

Table S4: Log Rank Test - Full Year

| Category | Procedure | 2015 Chi-Sq (P*>* Chi-Sq) | 2016 Chi-Sq  (P*>* Chi-Sq) | |
| --- | --- | --- | --- | --- |
| Predictable & | Hip & Knee | 20.14 | 0.91 | |
| Discretionary |  | (*< .*0001) | | (0.3396) |
| Predictable & Non-Discretionary | Childbirth | 419.55  (*< .*0001) | | 318.37  (*< .*0001) |
| Non-Predictable & | AMI & Stroke | 12.83 | | 7.99 |
| Non-Discretionary |  | (0.0003) | (0.0047) | |
| Falsification | Appendectomy | 0.67 | 0.30 | |
|  |  | (0.4136) | (0.5830) | |

Note: Table shows the results of the log-rank tests for the Kaplan Meier curves in manuscript Figure 1. This is a *chi*-squared distributed test statistic with *k* = 1 degrees of freedom that measures whether the Kaplan-Meier cumulative incidence curves of SEP and OEP members are similar.

Table S5: Utilization among Marketplace Members who Enrolled in the Special Enrollment Period (SEP) vs. Open Enrollment Period (OEP) in 2015 and 2016
(unadjusted hazard ratios)

| Category | Procedure | 2015  (95% CI) | 2016  (95% CI) |
| --- | --- | --- | --- |
| Predictable & | Hip & Knee | 1.36 | 1.07 |
| Discretionary | Replacement | (1.19 - 1.55) | (0.93 - 1.23) |
| Predictable & | Childbirth | 2.14 | 1.91 |
| Non-Discretionary |  | (1.98 - 2.30) | (1.77 - 2.05) |
| Non-Predictable & | AMI & Stroke | 1.18 | 1.16 |
| Non-Discretionary |  | (1.08 - 1.29) | (1.05 - 1.29) |
| Falsification | Appendectomy | 1.09 | 0.93 |
|  |  | (0.89 - 1.35) | (0.73 - 1.19) |

Note: Table shows the unadjusted Cox regression results and includes members who enrolled from January-December in each year.

Table S6: Utilization among Marketplace Members who Enrolled in the Special Enrollment Period (SEP) vs. Open Enrollment Period (OEP) in 2015 and 2016
(adjusted hazard ratios, restricted to 2015 states)

| Category | Procedure | 2015  (95% CI) | 2016  (95% CI) |
| --- | --- | --- | --- |
| Predictable & | Hip & Knee | 1.59 | 1.50 |
| Discretionary | Replacement | (1.39 - 1.82) | (1.28 - 1.75) |
| Predictable & | Childbirth | 1.84 | 1.61 |
| Non-Discretionary |  | (1.71 - 1.98) | (1.48 - 1.74) |
| Non-Predictable & | AMI & Stroke | 1.38 | 1.55 |
| Non-Discretionary |  | (1.26 - 1.51) | (1.38 - 1.75) |
| Falsification | Appendectomy | 1.08 | 0.98 |
|  |  | (0.87 - 1.33) | (0.74 - 1.27) |

Note: Table shows the Cox regression results comparing special enrollment period (SEP) to open enrollment period (OEP) members in each year, adjusted for age and sex. This analysis is restricted to members in the 16 states that the insurer offered Marketplace coverage to in both 2015 and 2016.

Figure S2: Time from Marketplace Enrollment to Utilization among Marketplace Members who Enrolled in the Special Enrollment Period (SEP) vs. Open Enrollment Period (OEP) before (2015) and After (2016) the SEP Eligibility Verification Policy (Kaplan-Meier cumulative incidence curves)

Predictable & Discretionary [Hip & Knee Replacement]


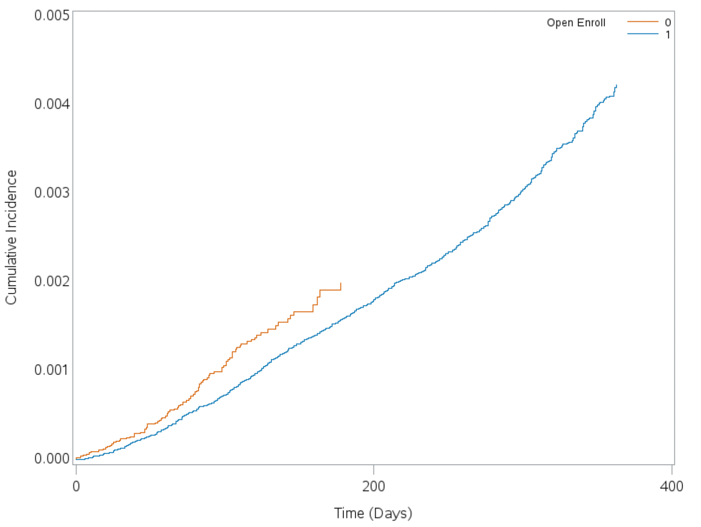

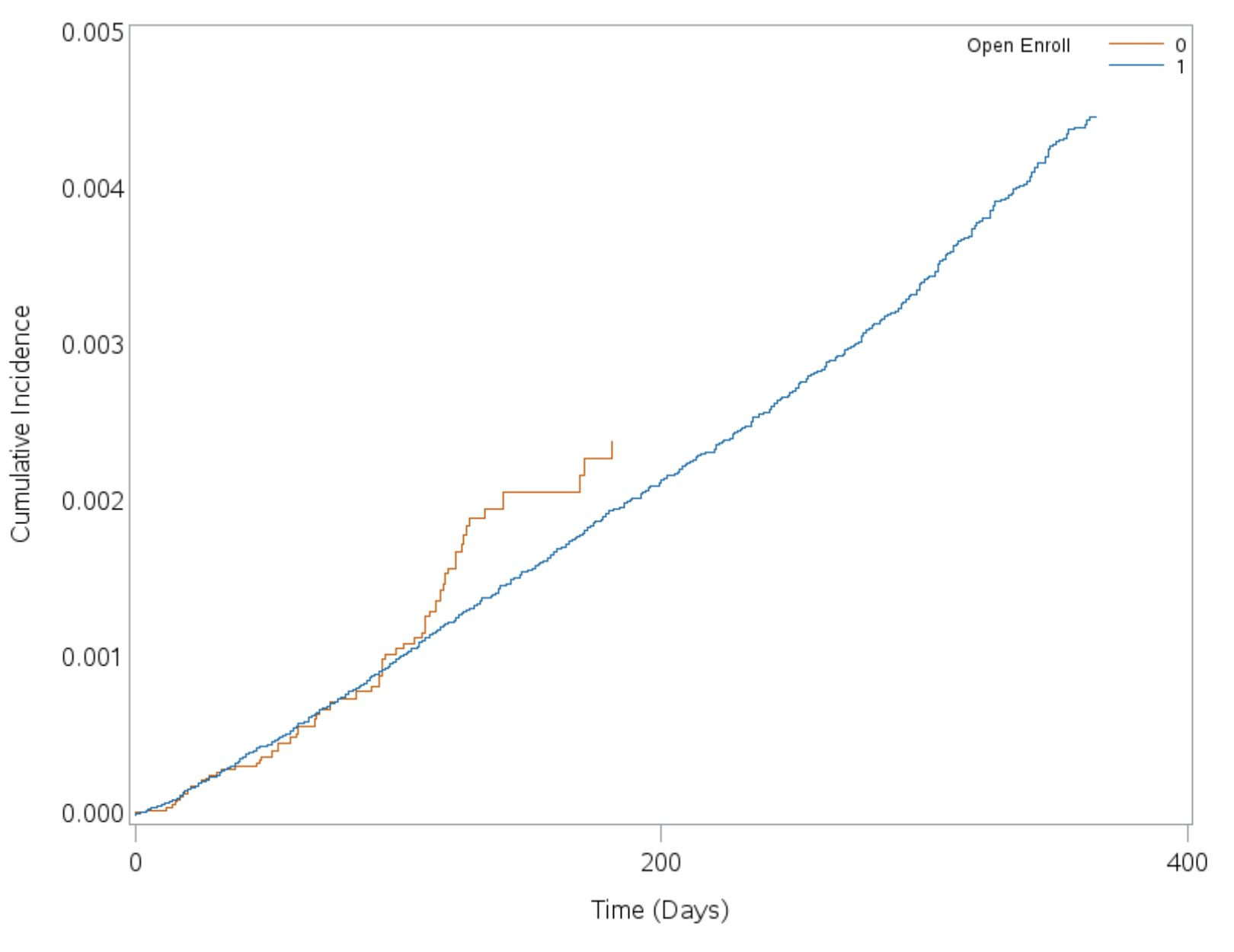


(a) 2015 (b) 2016

Predictable & Non-Discretionary [Childbirth]


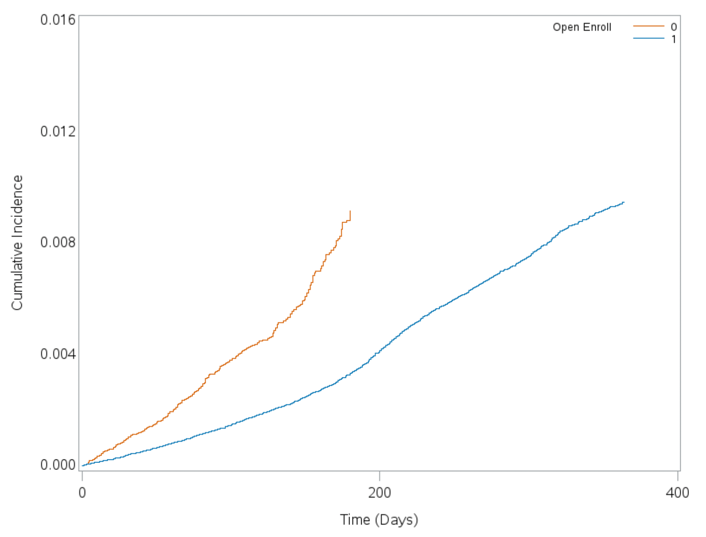

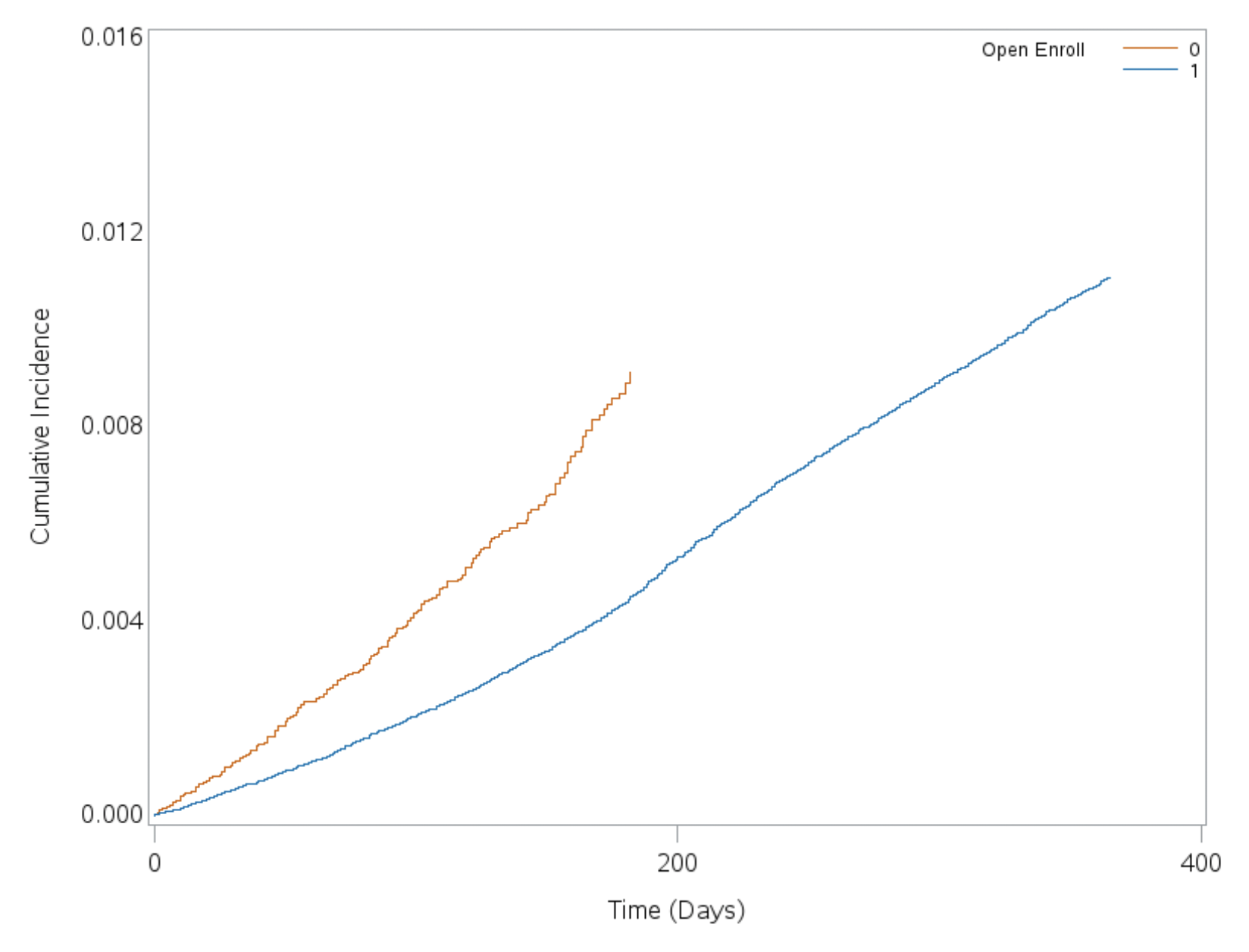


(c) 2015 (d) 2016

Non-Predictable & Non-Discretionary [AMI & Stroke]


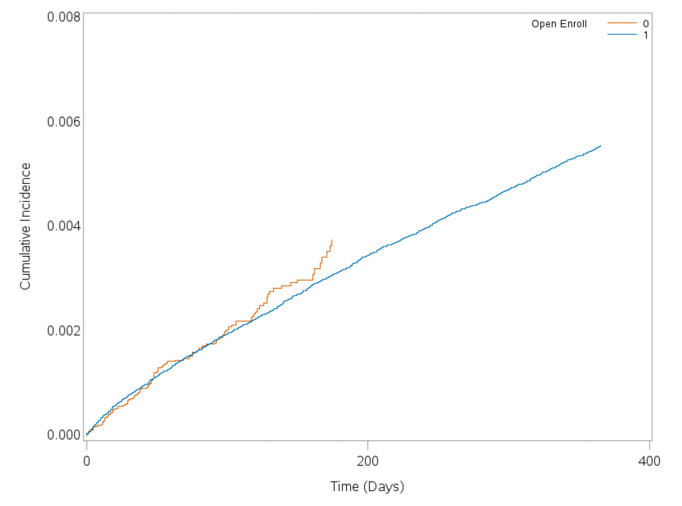

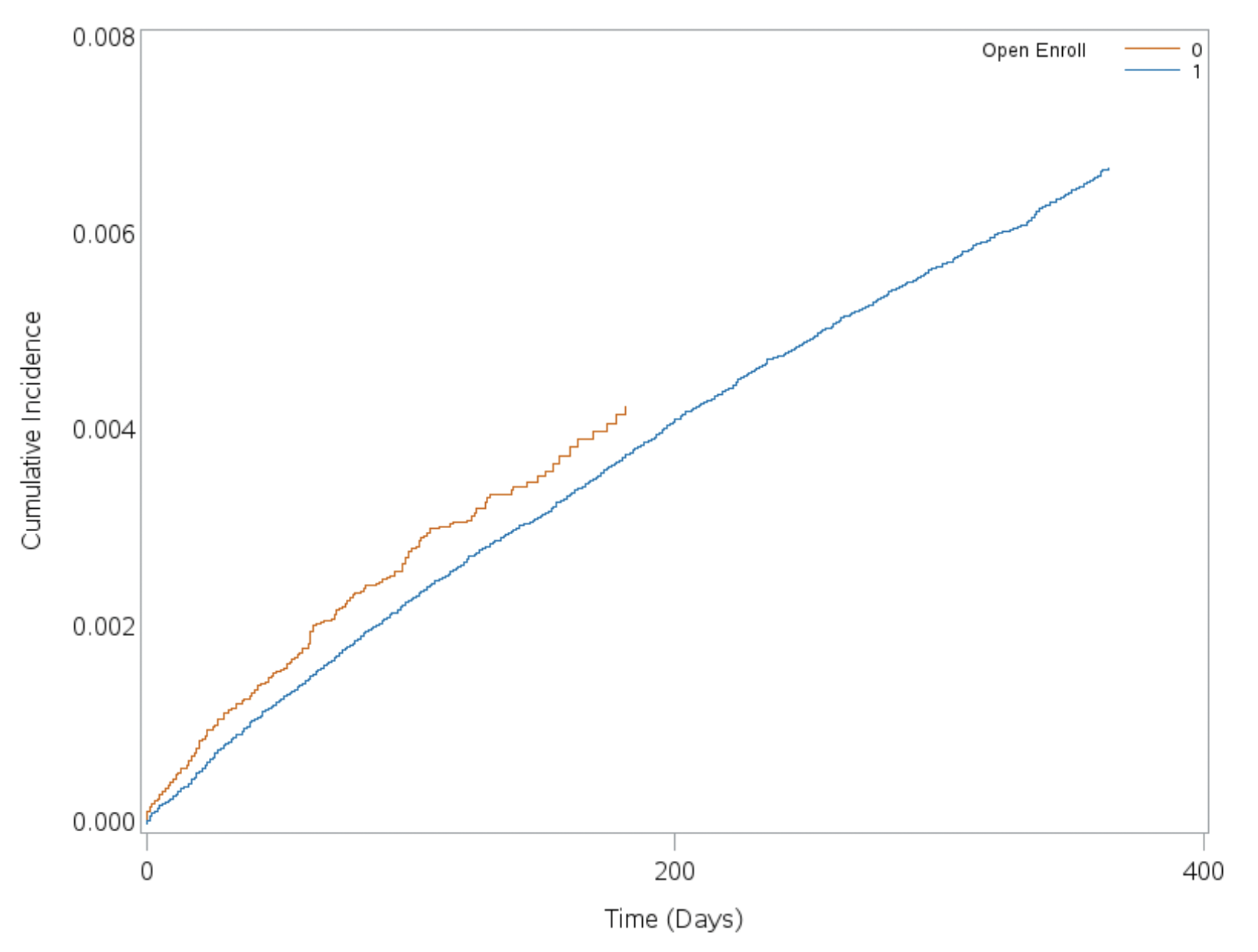


(e) 2015 (f) 2016

Predictable & Non-Discretionary [Appendectomy – Falsification Test]


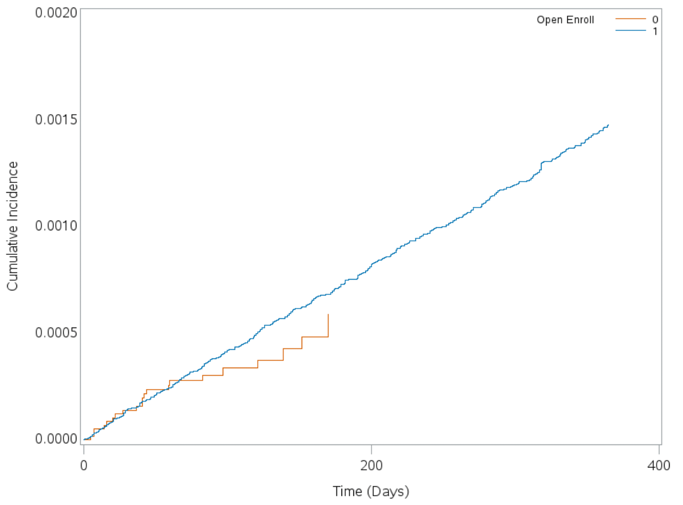

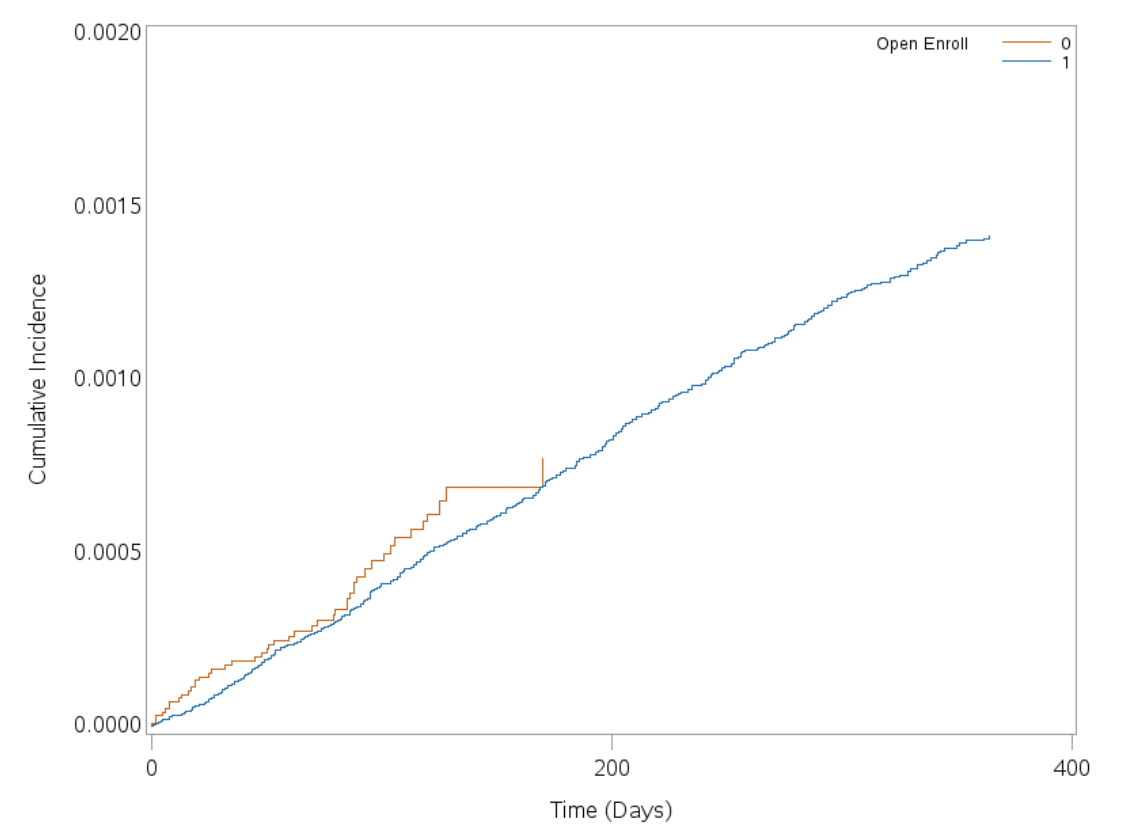


(g) 2015 (h) 2016

Note: Figures show the Kaplan-Meier cumulative incidence curves for SEP (orange) and OEP (blue) members. For each year, the results include SEP members who enrolled in July – December (i.e., after the implementation of the paperwork requirement policy in July 2016) vs. members who enrolled during the OEP. This analysis is restricted to members in the 16 states that the insurer offered Marketplace coverage to in both 2015 and 2016.

Table S7: Log Rank Test - Eligibility Verification Policy

| Category | Procedure | 2015 Chi-Sq (P*>* Chi-Sq) | 2016 Chi-Sq  (P*>* Chi-Sq) | |
| --- | --- | --- | --- | --- |
| Predictable & | Hip & Knee | 8.01 | 1.75 | |
| Discretionary |  | (0.0047) | | (0.1861) |
| Predictable & Non-Discretionary | Childbirth | 282.32  (*< .*0001) | | 115.12  (*< .*0001) |
| Non-Predictable & | AMI & Stroke | 5.19 | | 0.69 |
| Non-Discretionary |  | (0.023) | (0.4064) | |
| Falsification | Appendectomy | 0.76 | 0.84 | |
|  |  | (0.3841) | (0.3605) | |

Note: Table shows the results of the log-rank tests for the Kaplan Meier curves in Appendix Figure 1. This is a *chi*-squared distributed test statistic with *k* = 1 degrees of freedom that measures whether the Kaplan-Meier cumulative incidence curves of SEP and OEP members are similar. This analysis is restricted to members in the 16 states that the insurer offered Marketplace coverage to in both 2015 and 2016.

Table S8: Utilization among Marketplace Members who Enrolled in the Special Enrollment Period (SEP) vs. Open Enrollment Period (OEP) before (2015) and After (2016) the SEP Eligibility Verification Policy
(unadjusted hazard ratios)

| Category | Procedure | 2015  (95% CI) | 2016  (95% CI) |
| --- | --- | --- | --- |
| Predictable & | Hip & Knee | 1.35 | 1.18 |
| Discretionary | Replacement | (1.09 - 1.66) | (0.93 - 1.49) |
| Predictable & | Childbirth | 2.54 | 1.99 |
| Non-Discretionary |  | (2.26 - 2.84) | (1.75 - 2.27) |
| Non-Predictable & | AMI & Stroke | 1.17 | 1.08 |
| Non-Discretionary |  | (1.02 - 1.33) | (0.90 - 1.29) |
| Falsification | Appendectomy | 1.15 (0.84 - 1.57) | 0.81 (0.52 - 1.27) |
|  |  |  |  |

Note: Table shows the unadjusted Cox regression results in the years before (2015) and after (2016) the SEP eligibility verification policy change went into effect. For each year, the results include SEP members who enrolled in July – December (i.e., after the implementation of the paperwork requirement policy in July 2016) vs. members who enrolled during the OEP. This analysis is restricted to members in the 16 states that the insurer offered Marketplace coverage to in both 2015 and 2016.

Figure S3: Distribution of Days from Marketplace Enrollment to Childbirth for Marketplace Members who Enrolled in the Open Enrollment Period (OEP) vs. Special Enrollment Period (SEP) in 2015 and 2016


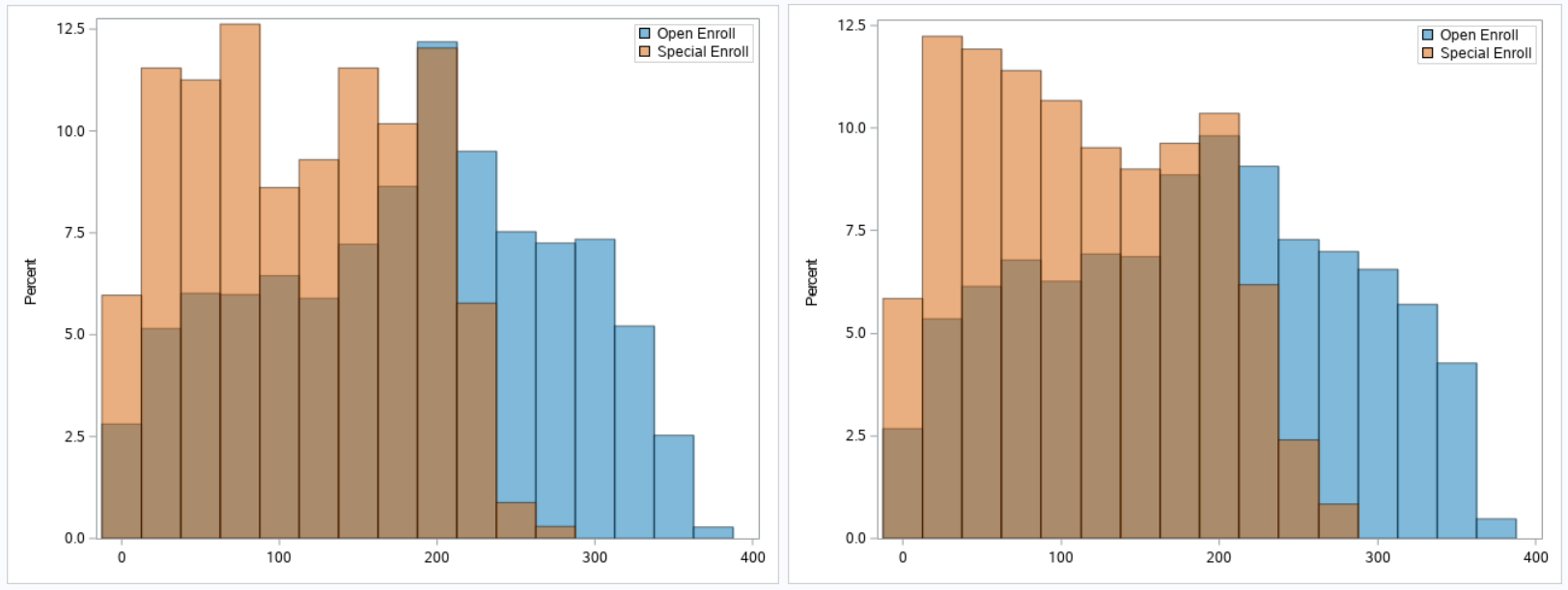


(a) 2015 (b) 2016

The mean (SD) number of days for SEP and OEP by year are:

- 2015: 113.7 (68.3) for SEP patients vs. 181.9 (92.9) for OEP patients.
- 2016: 113.4 (70.9) for SEP patients vs. 182.7 (96.7) in OEP patients.
